# Supplementary material for: Is short-term-variation of fetal-heart-rate a better predictor of fetal acidaemia in labour? A feasibility study
Source: PLoS One. 2020 Aug 3;15(8):e0236982. doi: 10.1371/journal.pone.0236982 (PMC7398510; doi:10.1371/journal.pone.0236982)
Supplement: Attachment — Submitted filename: responses to reviewers comments _PLOS ONE_June 2020.doc [file pone.0236982.s001.doc]

# Manuscript Ref: PONE-D-20-14044

# Title:” Is short-term-variation of fetal-heart-rate a better predictor of fetal acidaemia in labour? A feasibility study"

We thank the reviewers for painstakingly going through the manuscript, accepting it with positive feedback and suggesting some revisions.

**Response to Reviewers comments:**

| ***Reviewer 1*** | |
| --- | --- |
| **Comments** | **Responses** |
| This is a well-organized and clearly written manuscript. The problem, also well described by the authors, is the large attrition in the study population and consequently the very limited number of cases with acidemia most of which is mild and thus unlikely to have an association with adverse outcome. | Many thanks for this comment. |
| The results are of interest and contribute to a growing body of evidence that STV does not predict acidemia. This is an important finding because many clinical guidance are based on the assumption that metabolic acidemia does not exist when heart rate variability is within the normal range. | Many thanks for this comment. |
| The definition of acidemia is generous. A pH of 7.20 is not associated with adverse outcomes In fact in one very large Oxford -based study of gases, the mean umbilical artery pH at birth was 7.208. (Eur J Obstet Gynecol Reprod Biol 2013 Jun;168(2):155-60.) Many others report mean UA pH values around 7.24 to 7.28.  The reported IQR in this study indicates that there were probably only 5 cases with pH<7.12. Please provide the number and percentage of cases with pH< 7.10 and pH <7.0.  study? | We agree with the reviewer’s comments  The reviewer is correct and there are only five cases with pH<7.2. We have updated the description of the summary statistic for cord blood pH as follows:  “The range of cord blood pH was from 6.997 to 7.416. The prevalence of cord blood pH < 7.2 was 33.3% (21/63), 95% CI: 22.9%-45.6%, with 1.6% (1/63) having a pH less than 7.0 and 31.7% (20/63) having a pH less than 7.2 but greater than or equal to 7.1.” |
| Please temper your results to indicate that STV as measured by the Dawes algorithm over the last 60 minutes of tracing before birth did not discriminate the control group from the acidemia group as they were defined in this study where most of the acidemia was between 7.12-7.15. | Please see the response below. |
| This study found that in general STV and LTV increased over time. (Figure 3) I believe this is an analysis of all cases. This is an interesting finding, but the general objective of this study is to determine if the two subgroups were different. Please compare the two study subgroups regarding their change in STV and LTV. | The reviewer is correct; this analysis is based on all cases.  We have run some additional analysis for STV and LTV. In the linear mixed effects models we added an additional fixed factor for pH group and then the interaction between event and pH group was tested to investigate if any differences between events differed by pH group. Neither the interaction in the STV model (P=0.924) nor the LTV model (P=0.943) was statistically significant suggesting any differences between events did not differ by pH group. This is perhaps not surprising given the sample size.  The following text has been added to the statistical methods section:  “A second model was fit for STV and LTV with an additional fixed factor included for cord pH group. The interaction between event and cord pH group was then tested to investigate if any differences between events differed by cord pH group”.  And to the results section:  We found no statistically significant interactions between labour event and cord pH group for STV (P=0.924) and LTV (P=0.943). This suggests that any differences between labour events did not differ by cord pH. |
| Lines 193-194. This sentence is vague. What do you mean? Is the reliability and accuracy of the Dawes algorithm unmeasured? or unsatisfactory? Is the reliability and accuracy of the combination of the Dawes algorithm using Monica data unmeasured? or unsatisfactory? The Dawes algorithm and the Monica device are the basic “instruments” of your study. Do you lack confidence in the accuracy of this combination to measure variability? Please clarify. | We agree with the reviewer’s comments. This sentence is vague and raises concern regarding the validity of using Monica AN24.  This is not true as many studies have demonstrated the reliability and accuracy of using the Monica AN24 in measuring computerised FHR parameters using the Dawes algorithm, but the true clinical benefit during labour has yet to be determined.  The sentence is revised. |
| It is possible that the STV increases with mild acidemia and then falls with more extreme aberrations. Such a speculation cannot be examined without adequate representation of cases along the entire spectrum of pH measurements. Such a bimodal response would make comparison of means or ROC curves difficult to interpret because both very high and very low variability may be a predictor of acidemia. Confine your conclusions to precisely what your data can support. | We agree with the reviewer that if there is a bimodal response then it would be difficult to make comparisons in this way. We do not have data in this study to investigate this as the majority of births in the academia group have pH ≥ 7.1.  We have added some further text clarifying that our results are representative of the same we have and may not be representative of the wider population where there is a higher proportion of births with pH < 7.1. |
| **Reviewer 2** | |
| **Comments** | **Response** |
| **Sample Size:**  A reference should be provided for the sample size calculation using receiver operating characteristic curve (ROC) area under the curve (AUC) as the basis. Most Ob/Gyn readers, including this reviewer, are not familiar with this approach to sample size calculation. Presumably 'precision' is 95% confidence interval (CI). Please clarify. | The text describing the same size has been updated to reflect this comment and a reference has been added:  “The sample size was calculated to estimate the predictive potential for fetal acidaemia: with 200 participants and a prevalence of 10%, the area under the ROC curve of 0.8 could be estimated with a precision (half width of 95% confidence interval) of 0.12” |
| **Statistical Analysis:**  More detail on the manner of the analysis (regression?) should be included. The statistical computer package used for the regression, and non-parametric analyses, but particularly the ROC analysis, should be stated. | We have expanded the description of the analysis and included the statistical software used and the specific libraries for the ROC analysis and mixed effects models. This section now reads as:  “The diagnostic accuracy of STV for predicting arterial cord pH < 7.20 was assessed by calculating the area under the ROC curve along with the associated 95% confidence interval (CI). This analysis was repeated for the other computerised FHR (baseline FHR, LTV, accelerations and decelerations). In addition, Mann-Whitney U tests were used to compare FHR parameters between those with cord pH < 7.20 and those with cord pH ≥ 7.20.  The final analysis compared FHR parameters between different events during labour. For baseline FHR, STV and LTV, linear mixed effects models were used, while for the count variables of accelerations and decelerations, Poisson mixed effects models were used. In all cases, a fixed factor for labour event was included to estimate the effect of each event compared to the reference event of first measurement when the Monica was put on, and a random intercept for participant was included to allow for there being multiple measurements per individual. A second model was fit for STV and LTV with an additional fixed factor included for cord pH group. The interaction between event and cord pH group was then tested to investigate if any differences between events differed by cord pH group. The estimates from the linear mixed effects models are presented as the difference in mean and the estimates from the Poisson mixed effects models as incidence rate ratios (IRR).  Statistical analysis was conducted in R version 3.6.3 (ref) ROC analysis was conducted using the pROC library (ref), linear mixed effect models using the nlme library (ref), and Poisson mixed effects models using the lme4 library (ref). |
| **Results:**  In Figure 1, the flow diagram, the abbreviation 'PIS' is used and not defined. | Noted. Thanks  PIS is defined as “participant information sheet” in the list of abbreviation. |
| **Discussion:**  Line 240: ? typo 'declarations' should be 'decelerations' | Noted. Thanks.  This typo is changed to decelerations |
| Line 262: 'superiority for monitoring obese women' There is no data in this study to support this statement. If this is based on prior research, a reference should be provided. If true, this would be a very attractive feature. | Although the study data does not support this statement. There are studies on Monica AN24 which support this. We have referenced this statement. |
| **References:**  The citations for reference #20 and #27 are incomplete. | Noted. Thanks.  We have completed these references. |

We hope we have addressed your comment and we look forward for acceptance of this paper in your journal.

Yours sincerely

Dr Habiba Kapaya PhD, MRCOG
